# Targeting γ-aminobutyric acid pathways in irritable bowel syndrome: bridging central nervous system, enteric dysfunction, and the microbiota-gut-brain axis

**DOI:** 10.3389/fphar.2025.1677037

**Published:** 2025-12-22

**Authors:** Christian Lambiase, Francesco Rettura, Giusi Desirè Sciumè, Riccardo Tedeschi, Antonio Grosso, Lorenzo Cancelli, Andrea Bottari, Matteo Fornai, Luca Antonioli, Nicola de Bortoli, Massimo Bellini

**Affiliations:** 1 Gastrointestinal Unit, Department of Translational Research and New Technologies in Medicine and Surgery, University of Pisa, Pisa, Italy; 2 Regional Center for Functional and Motility Digestive Disorders, Azienda Ospedaliero Universitaria Pisana, Pisa, Italy; 3 Gastroenterology Unit, Annunziata Hospital, AOCS, Cosenza, Italy; 4 Unit of Pharmacology and Pharmacovigilance, Department of Clinical and Experimental Medicine, University of Pisa, Pisa, Italy

**Keywords:** GABA, irritable bowel syndrome, IBS, gut microbiota, gut-brain axis

## Abstract

Irritable bowel syndrome (IBS) is a complex and multifaceted disorder of the gut-brain interaction. Recent evidence suggests that γ-aminobutyric acid (GABA) may be involved in the development of IBS symptoms. Indeed, the GABAergic system exerts many gastrointestinal functions, such as modulation of visceral pain, intestinal motility, intestinal barrier integrity and immune response. GABA receptors and transporters are present and may influence intestinal functions at multiple levels: in the central nervous system, in the enteric nervous system and at the gut epithelial level. Furthermore, the gut microbiota is capable of producing GABA. This may also suggest a direct link between. intestinal microbiota composition and GABAergic tone within the microbiota gut-brain axis. Confirming the involvement of GABAergic dysregulation in IBS, altered GABA signaling and reduced GABA levels have been observed in this disease, especially in diarrhea-predominant subtypes. This review explores the possible roles of GABAergic dysregulation in IBS pathogenesis across multiple levels: in the central nervous system circuits, at the intestinal level, and in the microbiota-gut-brain axis interactions. Moreover, preclinical and limited clinical data regarding possible therapeutic approaches targeting the GABAergic system in IBS are discussed in the review. These include GABA receptor modulators, dietary supplements, probiotics producers of GABA and novel combinations such as GABA–Melissa officinalis. However, despite promising results, current evidence on these approaches is limited and mainly based on animal models. Therefore, randomized clinical trials are needed to establish the efficacy of GABA-based products in IBS management.

## 1 Introduction

Irritable bowel syndrome (IBS) is a chronic disorder of gut-brain interaction characterized by persistent or intermittent abdominal pain associated with altered bowel habits (Drossman and Hasler, 2016). It affects about 4% of the general population (Bellini et al., 2022), but its pathophysiology remains poorly understood (Bellini et al., 2014). It has been hypothesized that altered intestinal motility, food allergy/intolerance, enteric infection/inflammation, gut microbiota changes, altered intestinal immunity, epithelial barrier impairment, genetic and epigenetic factors, physiological, psychosocial, and environmental factors (Bellini et al., 2022; Bellini et al., 2014), could contribute in various ways to microbiota-gut-brain (MGB) axis dysregulation and the generation of IBS symptoms (Vasant et al., 2021). Also, neurotransmitter alterations could play an important role in IBS pathophysiology.

Recent research has suggested a possible role of γ-aminobutyric acid (GABA), an inhibitory neurotransmitter that exert many different functions throughout the body, in the development of IBS symptoms, particularly emphasizing its involvement in modulating visceral pain and the MGB axis interaction (Loeza-Alcocer et al., 2019). Indeed, the gastrointestinal (GI) tract of rat models has been found to contain multiple sources of endogenous GABA (Grider, 1998; Wang et al., 2006). GABA appears to be involved in visceral nociception, GI secretion and motility, modulation of colonic afferent excitability, strengthening of the epithelial barrier and modulation of the immune-inflammatory response and cytokine production (Loeza-Alcocer et al., 2019; Gros et al., 2021; Cataldo et al., 2020; Sokovic Bajic et al., 2019).

Is it therefore of great interest to review the current knowledge on the possible role of GABA in the pathogenesis and treatment of IBS. In this narrative review, we conducted a comprehensive online search without temporal restriction of PubMed (MEDLINE), Scopus, and Science Citation Index on the role of GABA in IBS, focusing on the central nervous system (CNS), enteric nervous system (ENS), and MGB axis.

## 2 Physiological highlights of GABA and its roles in the intestinal tract

GABA is the primary inhibitory neurotransmitter in the mammalian CNS (Wong et al., 2003). Several important physiological functions have been attributed to this small molecule, such as neurotransmission, induction of hypotension, diuretic effects, inhibition of oxytocin release, and relaxant effects (Hayakawa et al., 2004; Li et al., 2023).

GABA is produced through the α-decarboxylation of glutamate, operated by the enzyme glutamic acid decarboxylase (GAD) (Gros et al., 2021). GAD exists as two isoforms: GAD1 and GAD2. Ninety percent of the synthesized GABA is subsequently degraded by GABA-transaminase (GABA-T), present in both neurons and glial cells. After its release, GABA is taken up from the synaptic cleft by the GABA transporter (GAT) (Gros et al., 2021). Indeed, the clearance of extracellular GABA is not susceptible to enzymatic breakdown, but it is solely dependent on diffusion and uptake by the GAT, which can be expressed in several brain cell types, such as astrocytes and neurons (Drossman and Hasler, 2016). Empirical data suggest that the arrangement of GAT within the cell membrane is extremely dynamic and subject to activity-dependent changes (Scimemi, 2014; Whitworth and Quick, 2001).

GABA mediates its effects through binding with GABA receptor (GABA-R), which is either ionotropic (GABA-AR and GABA-CR) or metabotropic (GABA-BR) (Braat and Kooy, 2015; Hyland and Cryan, 2010). GABA-AR mediate fast synaptic transmission, while GABA-BR mediate slow synaptic transmission 18]. GABA-AR is a pentameric ligand-gated ion channel containing 19 different subunits (i.e., α1-6, β1-3, γ1-3, δ, ε, π, θ, and ρ1-3) (Olsen and Sieghart, 2009). Among the 19 subunits, π is the only subunit expressed in outside the CNS (Li et al., 2012). Their main role is to balance excitatory signals; their dysfunction can lead to neurological disorders and mental illnesses including epilepsy, memory impairment, schizophrenia, insomnia and anxiety (Kim and Hibbs, 2021).

On the other hand, GABA-BR belongs to class C of the G-protein coupled receptors (GPCRs), and it is a heterodimer formed by two subunits (i.e., GABA-B1R, and GABA-B2R) (Bettler et al., 2004). GABA-BR is a presynaptic receptor, and its roles involve the modulation of neurotransmitter release through the downregulation of Ca^2+^ influx via voltage-activated Ca^2+^ channels (Bowery et al., 2002). GABA-BR is associated with memory, mood and pain (Luscher et al., 2023; Koh et al., 2023). Despite the identification of GABA-CR, its role remains unclear (Lorenz-Guertin et al., 2023; Sarasa et al., 2020).

GABA has been identified throughout all the entire GI tract localized mainly in enteric nerves and in endocrine-like cells (Auteri et al., 2015). This suggests that at GI level, GABA may play a role both as a neurotransmitter and as an endocrine mediator, capable of influencing in both ways GI function (Hyland and Cryan, 2010). GABA-AR is present in the peripheral nerve terminals of the colon, and their stimulation by endogenous GABA contributes to the creation of the afferent excitability of the colon and visceral nociception (Loeza-Alcocer et al., 2019).

## 3 The role of GABAergic dysregulation in IBS

GABA and its pathophysiological role in IBS have been increasingly studied due to the central role of this inhibitory neurotransmitter in the MGB axis (Chen et al., 2022). In this regard, changes in the expression and function of GABA-R in the gut have been observed in IBS (Gros et al., 2021), and some studies have reported decreased levels of GABA in both serum and colonic tissues of IBS patients compared to healthy controls (Aggarwal et al., 2018), suggesting that the GABAergic pathway could be disrupted in IBS patients, contributing to the development of IBS symptoms.

A significant reduction in plasma GABA concentration in IBS-D patients compared to control subjects, an equal reduction in GAD2 expression was also observed, both at the mRNA and at protein levels. In contrast to expectations, the relative mRNA expression of GABA-degrading enzyme (i.e., GABA-T) was also reduced in IBS-D patients. However, when analyzing the difference between the fold change of GAD2 and GABA-T reduction, a tenfold greater reduction in GAD2 was observed compared to that of GABA-T reduction. The authors concluded that a significant reduction in GAD2 expression leads to an overall reduction in GABA levels in IBS-D patients despite the simultaneous decrease in GABA-T (Aggarwal et al., 2018).

**FIGURE 1:**
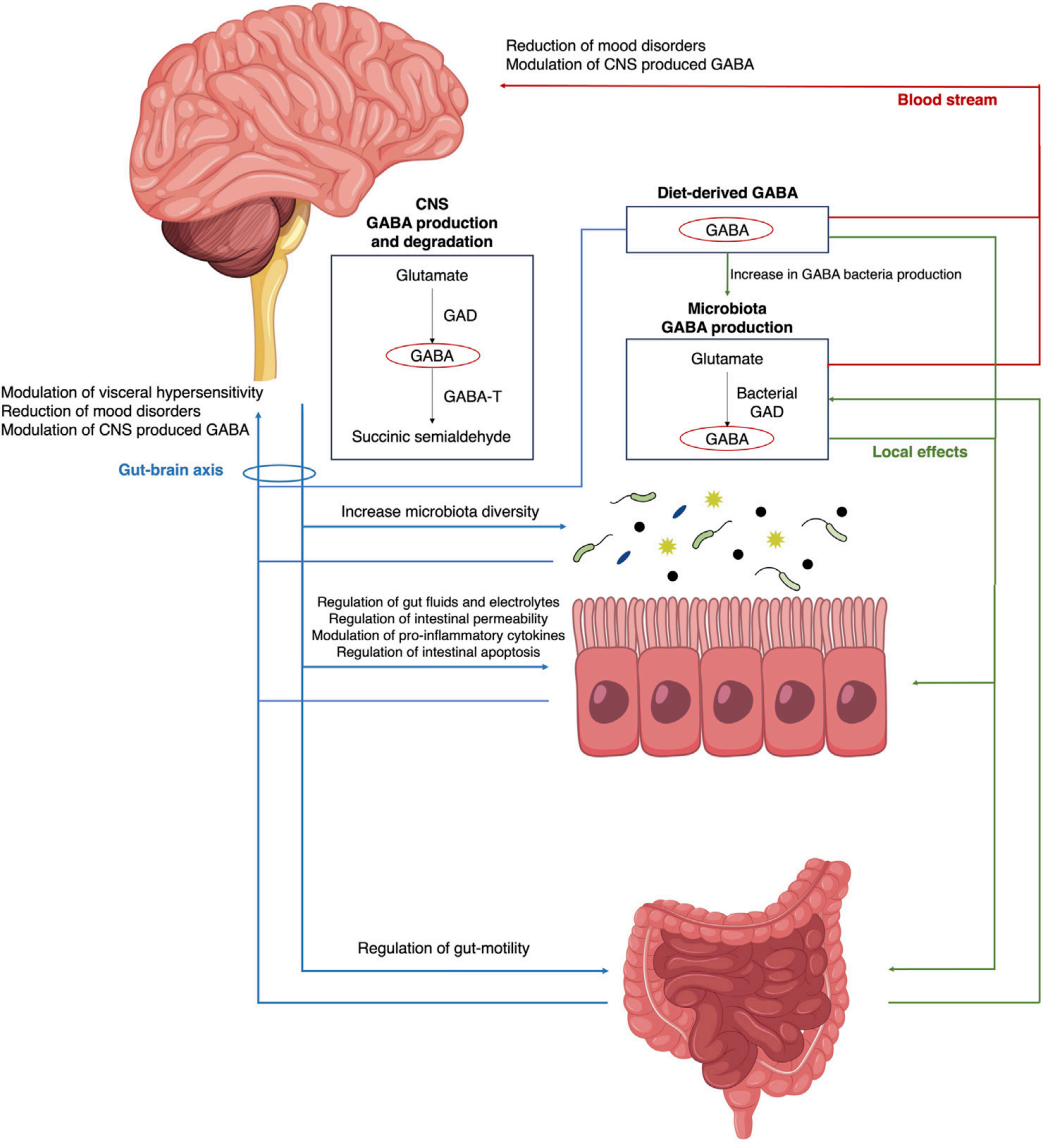
Production and roles of GABAergic transmission relevant for IBS pathophysiology. Abbreviations GABA, gamma-aminobutyric acid; CNS, central nervous system; MGB, microbiota-gut-brain; GAD, glutamate decarboxylase; GABA-T, GABA transaminase.

The possible disruption may involve the CNS, the enteric nervous system (ENS), the intestinal epithelial barrier (IEB), the gut microbiota, and the bidirectional communication between these elements (Figure 1).

### 3.1 Central nervous system (CNS)

GABA neurotransmission plays a crucial role in the CNS. Indeed, GABA can control the excitatory pathways in the CNS, and the loss of GABA-producing cells may alter the balance of excitation and inhibition (Guerriero et al., 2015). The balance between glutamatergic (excitatory) and GABAergic (inhibitory) tone is essential for normal neuronal functioning (Guerriero et al., 2015). Guo et al. demonstrated that the activation of GABAergic neurons projecting from the anterior cingulate cortex (ACC) to the lateral hypothalamic area (LHA) could elicit visceral hypersensitivity, intestinal motility dysfunction, and may produce anxiety-like behaviours in mouse models (Guo et al., 2024). Furthermore, these authors demonstrated that the activation of the GABAergic neurons led to an increase in histamine and serotonin (5-HT) levels in mice colonic tissues (Guo et al., 2024). These responses seem to be mediated by GABA-AR: the administration of Bicuculline (a GABA-AR antagonist), caused a reversion of these effects (Guo et al., 2024). Guo et al., in support of their studies, also reported that in their mice model of IBS, the chemogenetic inhibition of GABAergic neurons projecting from ACC to LHA was able to alleviate anxiety-like behaviours, improved visceral hypersensitivity and intestinal dysfunction (Guo et al., 2024). The central role of ACC in the genesis of IBS symptoms was also highlighted by Li et al., who confirmed that activation of GABAergic neurons in ACC led to a decrease in mechanical pain thresholds in naive mouse models (Guerriero et al., 2015). Furthermore, other studies confirmed that the activation of these GABAergic neurons was associated with anxiety-depression symptoms (Juarez-Salinas et al., 2019; Koga et al., 2018; Kantrowitz et al., 2021; Levar et al., 2017; Shao et al., 2021). The ACC is indeed closely associated with mood disorders related to GI disease and to visceral pain (Matisz and Gruber, 2022; Xiao et al., 2021), while the LHA is a brain region that responds to noxious stimuli, and plays a role in controlling pain-related behavioral responses (Guo et al., 2024). Given their important roles, these areas and their GABAergic connections may be altered by chronic abdominal pain and may be involved in symptom maintenance and in the genesis of psychological comorbidities, which have a higher prevalence in IBS (Fond et al., 2014). Supporting these hypotheses, patients with chronic pelvic pain appear to have lower levels of GABA in the ACC (Lançon and Séguéla, 2023).

Apart from the ACC-LHA GABAergic projections, other possible mechanisms involving GABAergic pathways in the CNS have been linked to IBS-like symptoms. In detail, Li et al. demonstrated, in a long-term water avoidance stress-induced IBS mouse model, the presence of increased GABAergic projections to the paraventricular nucleus (PVN) of the hypothalamus (Li et al., 2023). These GABAergic projections were able to inhibit the firing rate of neurons in the PVN and, as a consequence, decreased the expression and release of oxytocin (Li et al., 2023). Exogenous oxytocin has been demonstrated to improve gut motility and decrease abdominal withdrawal reflex scores. Therefore, inhibition of oxytocin by adaptive GABAergic projection in the PVN could be another important etiologic feature in IBS mediated by GABAergic dysregulation (Li et al., 2023).

Spinal GABAergic circuits modulate the brain’s processing of peripheral pain. GABA-AR can be divided into synaptic and extrasynaptic receptors based on their location in the synapse. Synaptic GABA-AR generally mediate classical phasic inhibition, whereas extrasynaptic GABA-AR produce tonic conductance when activated by low concentrations of GABA (Qian et al., 2023). Peripheral and spinal extrasynaptic α5-GABA-AR have been shown to play an important role in the regulation of pain pathways in a variety of chronic pain models. For instance, intrathecal administration of α5-GABA-AR inverse agonist L-655,708 attenuates chronic pain (Bravo-Hernández et al., 2016; Hernández-Reyes et al., 2019; Franco-Enzástiga et al., 2021). Therefore, extrasynaptic GABA-AR play distinct roles in pain pathways and further studies are needed.

Spinal GABA AR activation decreases spinal projection neuronal activity in a rat model of post-surgical pain, which was linked to less mechanical hypersensitivity but not to resting pain (Pradier et al., 2025). Pradier et al. discovered that the brain regions most impacted by spinal GABA-AR activation were those that received input via major ascending routes (i.e., the amygdala, the ventral posterior and ventromedial thalamus, and the hypothalamus), contrasting with observations in resting networks (Pradier et al., 2025). A better understanding of the modality-specific processing in the spinal cord between mechanical hypersensitivity and rest in the context of post-surgical pain could provide significant information for preventing its chronicity (Pradier et al., 2025), even in other chronic pain conditions such as IBS.

### 3.2 Enteric nervous system (ENS) and intestinal epithelial barrier (IEB): nociception, permeability and inflammation

GABA seems to play several roles at the intestinal level such as the control of motility and cell proliferation (Auteri et al., 2015). Most of its intestinal effects are mediated by the activation of GABA-AR or GABA-BR. GABA-ARs are widely expressed in the CNS and mediate fast neurotransmission, while GABA-BRs are predominantly expressed in the ENS and mediate a slow response (Hyland and Cryan, 2010). It is known that a functional GABAergic signaling system exists in colon epithelial cells (Li et al., 2012).

Regarding the first type of receptor, Seifi et al. demonstrated the expression of different GABA-AR subunits in neurochemically distinct cell types in the ENS of mice. Furthermore, the same group reported that different GABA-AR subtypes produced different and contrasting effects on the colon’s spontaneous contractility in mice (Seifi et al., 2014).

Since the ENS also plays an important role in regulating the local GI immune system, it is reasonable to hypothesize that this stress-induced change in GABA-AR-mediated ENS activity may also alter ENS-mediated immune function (Seifi et al., 2018). It has been reported that a significant contributing component to the pathophysiology of diarrhea-predominant IBS (IBS-D) is low-grade mucosal inflammation (Rana et al., 2012). In this regard, the activation of GABA-AR in the colon seems to be linked to intestinal inflammation. Indeed, mice that experienced stress early-life stress showed markedly changed colonic contractility and impaired barrier function mediated by GABA-AR (Seifi et al., 2018). Furthermore, in a mouse model, restraint stress led to colon inflammation. This effect could be mediated by the GABA-AR subunit α3 gene (*Gabra3*), as demonstrated by the fact that *Gabra3* expression and inflammation can be increased by stress in the colon of mouse (Seifi et al., 2018). Supporting this evidence, Seifi et al. reported that an α3-GABA-AR agonist was able to induce colonic inflammation *in vitro*, while α1/4/5-GABA-AR ligands were able to decrease the expression of inflammatory markers in the colon (Seifi et al., 2018). These mechanisms could be relevant to the pathogenesis of inflammatory bowel diseases (IBD).

Additionally, some studies have shown that eliciting GABAergic signaling, particularly through the activation of GABA-AR, reduced the overexpression of pro-inflammatory cytokines in HT-29 cells (a cell line from a white, female colorectal adenocarcinoma patient) stimulated by lipopolysaccharide (LPS) (Deng et al., 2023), increased the diversity of the gut microbiota (Seifi et al., 2018), and may modify the apoptosis of intestinal epithelial cells mediated by enterotoxigenic *Escherichia coli* (Xia et al., 2019), indicating a potential protective role in certain types of infective diarrhea.

Although less evidence is available regarding GABA-BR’s role in the MGB axis, it has been reported that its activation led to significant anti-inflammatory effects, that eased LPS-induced intestinal inflammation in mice (Seifi et al., 2014), a decrease in oxidative stress, an enrichment of the gut microbiota with increased levels of beneficial species (Deng et al., 2024), and a balance in excitatory pathways that may be associated to stress, anxiety or mood disorders (Cryan and Kaupmann, 2005). Furthermore, GABAergic neurons may influence gut motility through the modulation of smooth muscles in the gut, thereby affecting intestinal transit time (Hyland and Cryan, 2010). GABA also modulates the secretion of fluids and electrolytes in the intestine, has been shown that the GABAergic pathway is involved in transmitting signals of pain and discomfort from the gut to the brain (Hyland and Cryan, 2010). All these functions seem to be mediated by GABA-BR (Hyland and Cryan, 2010).

#### 3.2.1 Visceral hypersensitivity, nociception and permeability

The onset of abdominal pain has been linked with intestinal hypersensitivity. Some studies suggested that these alterations begin in the primary sensory neurons innervating the GI tract and reaching the CNS (Srinath et al., 2012). Since post-inflammatory visceral hypersensitivity is caused by enteric barrier and immune response alterations (Carabotti et al., 2015), a treatment capable of restoring the intestinal mucosa and of indirectly limiting inflammation and modulating the immune response could be the key. In this regard, GABA exerts multiple beneficial effects at the intestinal level by modulating immune-inflammatory response (Cataldo et al., 2020), acting also on cytokine production (Gros et al., 2021), and by strengthening IEB (Sokovic Bajic et al., 2019).

In a recent preclinical study, Lucarini et al. demonstrated that treatment with GABA-*Melissa officinalis* can counteract the onset and persistence of visceral hypersensitivity in a rat model of 2,4-dinitrobenzenesulfonic acid (DNBS) induced colitis (Lucarini et al., 2024). The treatment was able to limit damage to the colon, infiltration of tissue by polymorphonuclear cells (as demonstrated by decreased levels of myeloperoxidase), and oxidative stress (associated to a reduction of malondialdehyde levels) (Lucarini et al., 2024). Although the tested drug showed no significant effect on pro-inflammatory cytokine levels, GABA-*M. officinalis* combination helped restore the integrity of the IEB, as demonstrated by lower levels of lipopolysaccharide binding protein (LBP) and increased expression of claudin-1, and reduced the activation of both enteric and spinal glial cells, which is linked to chronic pain (Lucarini et al., 2024).

**FIGURE 2:**
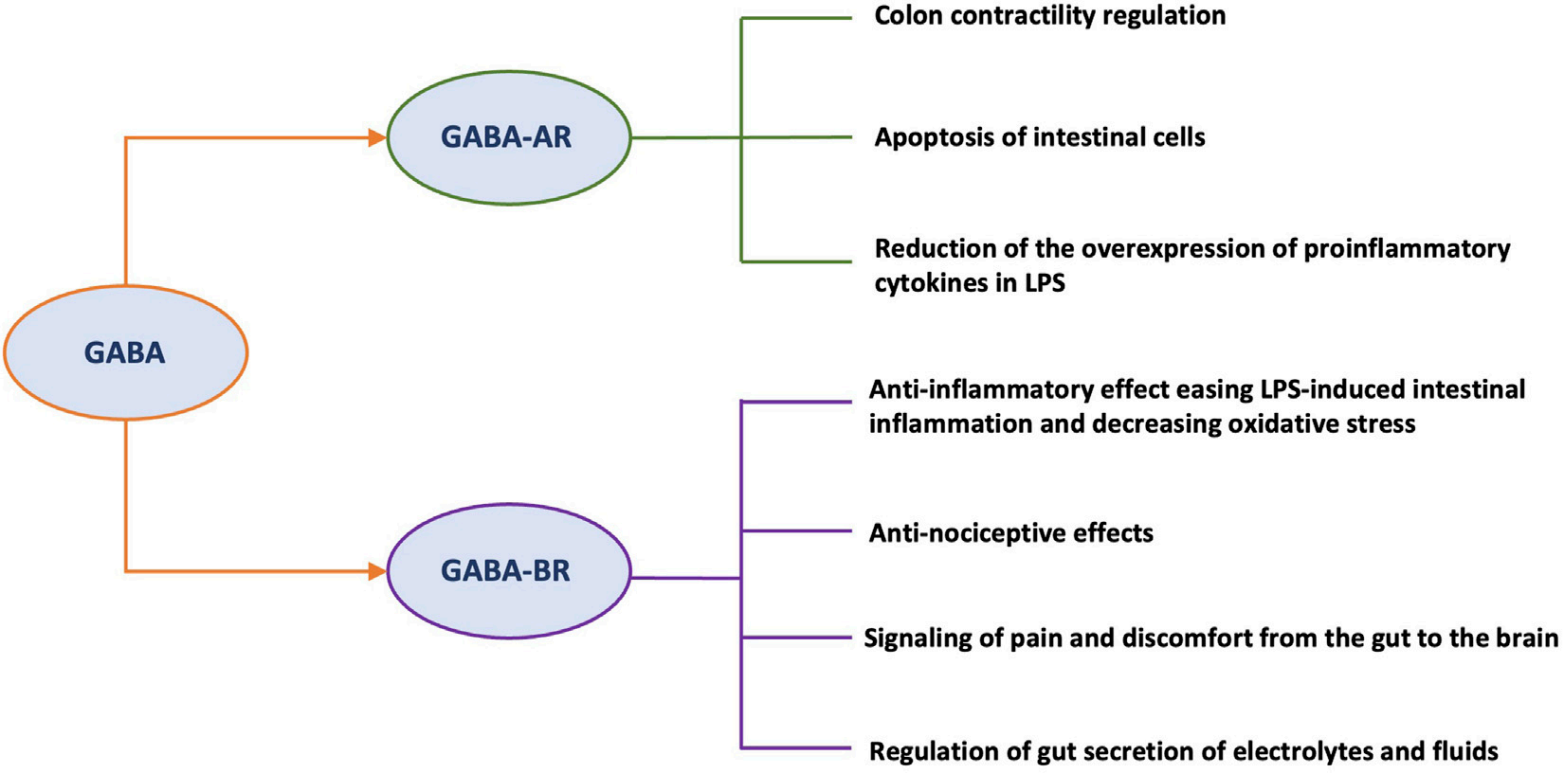
Roles of different GABA receptors relevant to the pathophysiology of IBS. Abbreviations: GABA, gamma-aminobutyric acid; GABA-R, GABA receptor; GABA-AR, GABA ionotropic receptor; GABA-BR, GABA metabotropic receptor; LPS, lipopolysaccharide; CNS, central nervous system; MGB, microbiota-gut-brain.

Post-inflammatory visceral hypersensitivity is also supported by the sensitization of nociceptors in the colon. As the main inhibitory neurotransmitter, GABA plays a key role in regulating pain signaling by acting directly on different types of receptors (i.e., GABA-AR and GABA-BR) (Hyland and Cryan, 2010) (Figure 2). In a preclinical study, diazepam was shown to decrease visceromotor response (VMR) in both dextran sodium sulfate (DSS)-treated and control mice by modulating of peripheral GABA-AR signaling (Loeza-Alcocer and Gold, 2022). When inflammation is present, the colon’s extracellular GABA levels drop (Aggarwal et al., 2017). In the CNS, inhibition of GABA-AR may contribute to inflammatory hypersensitivity. Hence, visceral pain might result from decreased GABA-mediated suppression of nociceptive signals from the colon directed to the CNS (Loeza-Alcocer and Gold, 2022). Furthermore, Coleman and Spiller reported that GABA-BR agonists may be a possible therapeutic target to alleviate pain in IBS patients (Coleman and Spiller, 2002). It was subsequently demonstrated that activation of GABA-BR produces anti-nociceptive effects in a rat model of mechanically induced visceral pain (Brusberg et al., 2009).

There is increasing interest also towards non-coding RNAs. Several non-coding RNAs many microRNA have been identified as involved in the pathophysiology of IBS (Chen et al., 2024). Among these, the miR-19 family were implicated in both visceral hypersensitivity and augmented intestinal permeability in IBS-D patients (Dothel et al., 2023). Chai et al. detected three tRNA-derived small RNAs, that were highly expressed in intestinal biopsies from IBS-D patients compared to healthy controls, which were related to the clinical symptoms of IBS-D (i.e., degree of abdominal pain, abdominal distension, and stool morphology). These differential tRNA-derived small RNAs, beyond representing potential biomarkers of IBS-D, have been shown to be potentially involved in some key signaling pathways, such as GABAergic synapse and TNF-α (Chai et al., 2021). However, additional studies are needed to expand the role of non-coding RNAs and identify enzymes and signaling paths involved in IBS complexity.

#### 3.2.2 Inflammation and permeability in diarrhea-predominant IBS (IBS-D) and the role of GABA

GABA neurotransmitter alteration in IBS-D patients may play a role also in inflammatory processes (Talley, 2001; Crowell, 2004). Lucarini et al., through the administration of GABA-*M. officinalis* in a rat model of post-inflammatory IBS, observed a reduction of post-inflammatory visceral pain (Lucarini et al., 2024). Linking inflammation to visceral hypersensitivity, Aggarwal et al. demonstrated that diminished levels of circulating GABA and altered GABAergic signaling may contribute also to the pathogenesis of IBS-D by interfering with inflammatory processes (Aggarwal et al., 2018). They observed a downregulation of both GABA-BR subunits (i.e., GABA-B1R, and GABA-B2R) in IBS-D patients compared to healthy controls (Aggarwal et al., 2018). Furthermore, authors demonstrated that GABA-BR function differed between patients with IBS-D and UC, reflecting the distinct inflammatory profiles of these two disorders (Aggarwal et al., 2017). The same authors in a different study showed an increased expression of GABA-B2R in colonic mucosal biopsies of UC patients (Aggarwal et al., 2017). It was found that another reason for the reduced GABA levels in IBS-D patients was the increased expression of the GABA transporter 2 (i.e., GAT-2) (Aggarwal et al., 2018), involved in GABA uptake and subsequent reduction of local GABA concentration. GAT-2 overexpression has also been reported during inflammatory conditions in patients with both multiple sclerosis and UC (Paul et al., 2014).

IBS-like symptoms (e.g., abdominal pain and changes in bowel movements) are also reported in patients before the diagnosis of IBD or during remission from inflammatory or infectious diseases (Spiller et al., 2016) and low GABA levels have previously been reported in serum of patients affected by UC (Aggarwal et al., 2017).

A mild inflammation has been linked also to the pathogenesis of IBS-D, as demonstrated by the increased expression of some proinflammatory cytokines in these patients (e.g., IL-1β, TNF-α and IL-6) and increased expression of inducible nitric oxide synthase (iNOS) (Aggarwal et al., 2018; Rana et al., 2012; An et al., 2016). Several studies have highlighted a possible anti-inflammatory role of GABA through the inhibition of the expression of inflammatory mediators (Aggarwal et al., 2018; Bhat et al., 2010; Yang et al., 2014). In an *in vitro* study on HT-29 cells stimulated through LPS, GABA administration was able to reduce the expression of proinflammatory cytokines mRNA (i.e., IL-1β, TNF-α, and IL-8), while bicuculline methiodide (a GABA antagonist) increased their expression (Aggarwal et al., 2018). A study conducted by Han et al. also reported that GABA can significantly inhibit the expression of TNF-α, IL-1β, and iNOS mRNA in LPS- stimulated RAW 264.7 cells (Han et al., 2007). A reduction in IL-1β production has also been demonstrated following treatment of LPS-activated peritoneal macrophages (purified from mice) with GABA-AR agonist (Bhat et al., 2010). Yang et al. also suggested that TNF-α production decreased in lipid-loaded human monocyte-derived macrophages treated with GABA and topiramate (Yang et al., 2014).

### 3.3 Microbiota-gut-brain (MGB) axis

The CNS communicates with the ENS, muscle layers, and gut mucosa. This bidirectional communication modulates intestinal motility, immunity, permeability and secretions (Carabotti et al., 2015). In the last decade, researchers have shown that this bidirectional communication is further complicated by its interaction with the gut microbiota, so today the concept of the MGB axis has been introduced (Carabotti et al., 2015). Indeed, it is well documented that alterations in the MGB axis affect both the IEB and the integrity of the blood-brain barrier, playing an important factor in the pathogenesis of both GI diseases (such as IBS) and CNS diseases (Benvenuti et al., 2024; Pittayanon et al., 2019; Bellini et al., 2024; Bellini et al., 2023). There are five main ways in which the gut microbiota and the brain can communicate: the gut–brain neural network pathways, the hypothalamic–pituitary–adrenal axis, the gut immune system, the neurotransmitters synthesized directly by the gut microbiota (e.g., GABA), and the barrier pathways (i.e., IEB and blood-brain barrier) (Dumitrescu et al., 2018; Strandwitz, 2018). The communication through all these components can be bidirectional, so GI signals can influence the CNS and CNS signals can alter GI function. Overall, the CNS can interfere with the intestinal microenvironment by modulating intestinal motility and neuroendocrine pathways (Gao et al., 2020; Varatharaj and Galea, 2017; Stasi et al., 2019; Stasi et al., 2012). On the other hand, the gut microbiota plays a pivotal role in the MGB axis due to its interactions with the immune system, IEB and/or ENS-vagus nerve pathways (Varatharaj and Galea, 2017; Stasi et al., 2019). Certain intestinal bacterial species can directly stimulate enterochromaffin cells to produce various neurotransmitters (e.g., 5-HT), or neuropeptides (e.g., peptide YY, neuropeptide Y, cholecystokinin, glucagon-like peptides-1 and -2, and substance P) (Stasi et al., 2017; Liu et al., 2020). Moreover, specific bacterial metabolites (e.g., short-chain fatty acids, vitamins or neurotransmitters, such as acetylcholine, dopamine, norepinephrine, GABA or 5-HT) can pass across the IEB, flow into the bloodstream and reach the CNS (Carabotti et al., 2015; Gao et al., 2020; Chen et al., 2021).

In IBS, GABA can affect gut function and brain activity in IBS, assisting in regulating their connection (Azarfarin et al., 2024). Patients with IBS show differences in brain activation in response to visceral pain compared to healthy subjects; this suggests that patients with IBS lack of central activation of descending inhibitory pathways, possibly due to a decrease in GABA-mediated suppression (Aggarwal et al., 2017; Tanaka et al., 2018). GABA has a significant impact on how the brain interprets and reacts to stress and emotions, which are prevalent in IBS and can affect the onset and intensity of GI symptoms (Bravo et al., 2011).

GABA circuits, receptors, and signaling are significantly affected by the gut microbiota, which could therefore modulate neurochemical pathways involved in the IBS pathophysiology (Rajilić-Stojanović et al., 2015). Both prokaryotic and eukaryotic cells are able to produce GABA through the GAD-mediated decarboxylation of glutamate (Strandwitz, 2018; Mazzoli and Pessione, 2016). Both Gram-positive and Gram-negative bacteria express GAD, which is related with pH homeostasis and metabolic energy synthesis (Tsai et al., 2013).

In detail, food-derived lactobacilli and gut-derived *Lactobacillus* and *Bifidobacterium* species were able to biosynthesize GABA *in vitro* (Barrett et al., 2012; Yunes et al., 2016). Furthermore, a recent *in vitro* study using 13 bacterial strains, including *Levilactobacillus brevis*, *Lactiplantibacillus plantarum*, *Lacticaseibacillus paracasei*, *Ligilactobacillus salivarius*, and *Streptococcus thermophilus* species, was able to change the gut-microbiota composition by increasing the abundance of *Veillonellaceae* and *Bacteroides*, two potential GABA producers that have been related to anti-inflammatory effects (Casertano et al., 2024).

Further evidence supporting the crucial role of the gut microbiota was provided by the discovery that GABA levels were significantly reduced in both the feces and blood of germ-free mice (Matsumoto et al., 2017). Similarly, antibiotics can alter fecal levels of GABA (Fujisaka et al., 2018). Long-term administration of *Lactobacillus rhamnosus* JB-1 in a murine model has been shown to reduce anxiety-like and depressive behaviors (Bravo et al., 2011). This was associated with changes in the expression of GABA receptors in specific areas of the brain (Bravo et al., 2011). Moreover, a more recent study reported an increase in brain GABA in mice after supplementation with *Lactobacillus rhamnosus* JB-1 (Janik et al., 2016). From a mechanistic perspective, it has been suggested that the vagus nerve is a significant route whereby beneficial bacteria in the gut could influence behaviours related to anxiety and depression (Bravo et al., 2011). Conversely, vagotomy has been shown not invariably suppress behavioural changes induced by gut microbes (Bercik et al., 2011).

In this regard, it is interesting the development of psychobiotics, a novel category of probiotics with beneficial effects capable of influencing brain function by acting on immune responses, hormonal signaling as well as modulating neurotransmitter levels and bioavailability (Kundu et al., 2017).

Dysbiosis which causes an alteration in bacterial metabolism and interfere with the IEB, emerges as a common feature of painful GI diseases (e.g., IBS), both in preclinical and clinical studies (Guo et al., 2019; Lucarini et al., 2022; Shaikh et al., 2023). Based on these pathophysiological data, some studies have evaluated the efficacy of GABA-producing probiotic strains in modulating visceral nociception, mainly in preclinical settings (Pokusaeva et al., 2017; Laroute et al., 2022; Jin et al., 2023; Gomes et al., 2025). The human gut commensal *Bifidobacterium dentium* produces a significant quantity of GABA, and oral supplementation has been shown *in vivo* to regulate sensory neuron activity in a rat fecal retention model of visceral hypersensitivity (Pokusaeva et al., 2017). Moreover, a selenium-enriched *Bifidobacterium* (Se-*B. longum* DD98) was shown to act on the MGB axis by relieving the intestinal symptoms of IBS, reducing intestinal permeability and inflammation, and regulating mood-related behaviours in a mice model of stress-induced IBS. This was associated with the upregulation of 5-HT, GABA, neuropeptide Y, and brain-derived neurotrophic factor, which are indicators closely related to mood and MGB axis (Jin et al., 2023).

Laroute et al. demonstrated that *L. lactis* NCDO2118, a high GABA producer, exerts visceral anti-hypersensitivity effects due to its high GAD activity in an acute stress IBS-like rat model. Conversely, *L. lactis* NCDO2727, a low-level GABA-producing strain, despite similar genes for GABA metabolism, had no antinociceptive effect *in vivo*, nor did the NCDO2118 strain when unable to produce GABA. The beneficial effect observed for *L. lactis* NCDO2118 was therefore attributed to the production of GABA in the GI lumen and its subsequent stimulation of the GABA-BR (Laroute et al., 2022). The same group showed that *L. lactis* CNCM I-5388 had higher intracellular GAD activity, resulting in increased GABA production under the same *in vitro* conditions as strain NCDO2118. Additionally, *L. lactis* CNCM I-5388 showed greater anti-visceral hypersensitivity efficacy both in terms of onset of action and partial persistence of effects after 5-day treatment interruption in the same acute stress rat model compared to strain NCDO2118 (Gomes et al., 2025). Finally, the same group showed that anti-visceral hypersensitivity properties were also mediated by ethanol-inactivated Lactococcus lactis CNCM I-5388 through an active GAD enzyme, resulting in increased GABA levels in the stomach in the same IBS rat model (Gomes et al., 2025). These preclinical findings in animal models suggest that GABA-producing bacteria in both viable and non-viable forms might represent a potential treatment for alleviating IBS symptoms in humans as well, especially considering their effect on visceral hypersensitivity. Therefore, the gut microbiota may play an important role in positively modulating the stress response, and visceral GI nociception by acting on the host’s GABAergic signalling (Mazzoli and Pessione, 2016; Molon et al., 2016; Mayer et al., 2014; Strandwitz et al., 2019). However, clinical evidence is still lacking.

## 4 Gabaergic agents as a possible therapeutic approach in IBS

The management of IBS remains a challenge for physicians. Conventional treatments for IBS primarily target predominant stool pattern (e.g., opioid agonists such as loperamide, and laxatives such as polyethylene glycol) offering limited efficacy in addressing symptoms like bloating and abdominal pain (Black et al., 2020; Black et al., 2018). At present, there are several management strategies for IBS, including dietary approaches (e.g., low Fermentable Oligo-, Di-, Mono-saccharides, and Polyols diet), antispasmodic agents, antihistamines (e.g., ebastine), 5- HT3 antagonists (e.g., ondansetron), mixed opioid agonists/antagonists (i.e., eluxadoline), bile acid sequestrants (e.g., colestyramine), ileal bile acid transporter agents (e.g., elobixibat), microbiota-modulating treatments (e.g., prebiotics, probiotics, postbiotics, non-absorbable antibiotics, and fecal microbiota transplantation), mucoprotectants, gut-brain neuromodulators (e.g., TCAs, SSRIs, and SNRIs), brain-gut behavioral treatments (e.g., cognitive behavioral therapy), and psychological therapies (Vasant et al., 2021; Lembo et al., 2022; Savarino et al., 2022; Lambiase et al., 2024; Bellini et al., 2021; Chang et al., 2022; Rettura et al., 2025). However, these therapies frequently produce only partial and inadequate effects (Lucak et al., 2017), most likely because to the complicated and yet poorly understood pathophysiology of IBS. The increased knowledge of the role of the GABAergic signaling system in IBS could offer opportunities for innovative gut-brain neuromodulators. For instance, targeting GABA receptors with specific agonists or modulators could provide new perspectives for managing abdominal pain and psychological symptoms associated with IBS (Mayer et al., 2015).

We have several drugs acting through the modulation of GABA that could potentially be useful for IBS treatment (Table 1). Gabapentin and pregabalin (generally used to treat neuropathic pain) are two α_2_δ auxiliary protein ligands of voltage-gated calcium channels. Their full mechanism of action is not yet understood. Although they do not bind to GABA receptors and have no effects on GABA production or degradation, they are structurally similar to GABA (Senderovich and Jeyapragasan, 2018). Several studies have demonstrated the anti-hyperalgesic effects of gabapentin and pregabalin on more severe GI symptoms (Saps and Miranda, 2017; Drossman et al., 2018). Gabapentin was able to improve pain and anxiety in mice, and reduced the cerebral nociceptive response to colorectal distension, but its use is limited by its side effects (i.e., hepatotoxicity and neurotoxicity) (Zhang et al., 2014). In a clinical study, forty patients with IBS-D were randomly assigned to a 5-day treatment with gabapentin (i.e., 300 and 600 mg/day), observing an increase in rectal sensory thresholds resulting from a reduction in rectal sensitivity to distension and enhanced rectal compliance (Lee et al., 2005). Pregabalin, for its analgesic and anxiety-relieving effects, was approved by FDA for the treatment of fibromyalgia and neuropathic pain (Cross et al., 2025). Pre‐clinical studies suggest that pregabalin is effective visceral analgesic (Diop et al., 2002; Eutamene et al., 2000; Ravnefjord et al., 2008; Million et al., 2007). Houghton et al. showed that pregabalin increased distension sensory thresholds (i.e., first sensation, desire to defecate, and pain) to normal levels in IBS patients with rectal hypersensitivity. In addition, the authors reported that pregabalin was generally well tolerated, except for mild dizziness and somnolence (Houghton et al., 2007). It was subsequently shown that pregabalin did not reduce colonic pain related to distension in patients with IBS-C (Iturrino et al., 2014).

**TABLE 1:** Medications potentially affecting GABA pathways divided in clinical and preclinical studies.

Medications	Mechanism of action GABA related	Preclinical study on GI effects	Clinical study on GI effects	References
*Gabapentin*	Structurally related to GABA. Binds the α_2_δ auxiliary protein of voltage-gated calcium channels	It reduces the brain’s nociceptive response to colorectal distension in mice	It increases rectal sensory thresholds resulting from a reduction in rectal sensitivity to distension and enhanced rectal compliance in IBS-D patients	Zhang et al. (2014), Lee et al. (2005)
*Pregabalin*	Structurally related to GABA. Binds the α_2_δ auxiliary protein of voltage-gated calcium channels	It reduces TNBS-induced colonic allodynia in the rat It suppress LPS-induced hyperalgesia in a rat model It reduces visceral pain and prevents spinal neuronal activation in rats It reduces the viscerosomatic and autonomic responses associated with visceral pain induced by colorectal distension and increases colonic compliance in rats	It increases distension sensory thresholds to normal levels in IBS patients with rectal hypersensitivity It does not reduce colonic pain related to distension in IBS-C patients It improves abdominal pain, bloating and diarrhoea in patients with IBS-D and IBS-M, but not in IBS-C patients	Diop et al. (2002), Eutamene et al. (2000), Ravnefjord et al. (2008), Million et al. (2007), Houghton et al. (2007), Iturrino et al. (2014), Saito et al. (2019)
*PD-217014*	Structurally related to GABA. Binds the α2δ auxiliary protein of voltage-gated calcium channels	It is effective in inhibiting visceral hypersensitivity in the rat TNBS model	It has no significant efficacy compared to placebo in reducing abdominal pain/discomfort in IBS patients in an RCT.	Ohashi et al. (2008), Houghton et al. (2025)
*Topiramate*	GABA-activated chloride channels	Amelioration of macro- and microscopic GI inflammation score in animal model with IBD.		Dudley et al. (2011)
*Diazepam*	Modulation of peripheral GABA-AR signalling	Decrease of VMR in both DSS-treated and control mice		Loeza-Alcocer and Gold (2022)
*Baclofen CGP7930*	GABA-BR agonist GABA-BR positive allosteric modulator	Reduction of visceral pain in rats		Brusberg et al. (2009)
*Melissa officinalis*	Preventing GABA metabolism	Counteracting the establishment and persistence of visceral hypersensitivity in a rat model of colitis induced by 2,4-DNBS. Direct modulation of inflammation and nociception. It contributes to restore the IEB integrity, as evidenced by LBP levels and higher expression of claudin-1, and to reduce both enteric and spinal glial cell activation, linked to pain chronicity.		Lucarini et al. (2024)
*GABA producing probiotics*	Direct action on GABA-R	*B. dentium* ATCC 27678 modulates sensory neuron activity in a rat fecal retention model of visceral hypersensitivity.In a mice model of stress-induced IBS, selenium-enriched *B. longum* DD98 shows to upregulate GABA levels and alleviates intestinal symptoms, reducing intestinal permeability and inflammation, and regulating mood-related behaviours *L. lactis* NCDO2118 exerts antinociceptive properties in an acute stress IBS-like rat model due to its GAD activity and GABA-BR stimulation L. lactis CNCM I-5388 exhibits higher intracellular GAD activity, resulting in increased GABA production and enhanced anti-visceral hypersensitivity efficacy in the same acute stress rat model as NCDO2118 strain The postbiotic *L. lactis* CNCM I-5388 with an active GAD enzyme increases GABA levels within the stomach and exhibits anti-visceral hypersensitivity properties in a rat model of IBS.	*Bifidobacterium adolescentis* PRL2019 reduces the severity and frequency of symptoms in children with IBS-C	Pokusaeva et al. (2017), Laroute et al. (2022), Jin et al. (2023), Gomes et al. (2025), Giorgio et al. (2025)
*GABA containing foods*	Direct action on GABA-R	Functional foods containing GABA have demonstrated antioxidant properties *in vitro*		Minerv et al. (2009), Linares et al. (2016), Park et al. (2005), Ramos et al. (2025)

Abbreviations: GABA, γ -aminobutyric acid; GAD, glutamic acid decarboxylase; IBS, irritable bowel syndrome; IBS-D, diarrhea-predominant IBS; IBS-M, mixed stool pattern IBS; GI, gastrointestinal; IBD, inflammatory bowel disease; VMR, visceromotor response; DSS, dextran sodium sulfate; DNBS, 2,4-dinitrobenzenesulfonic acid; IEB, intestinal epithelial barrier; LBP, lipopolysaccharide binding protein; LPS, lipopolysaccharide; RCT, randomised clinical trial; TNBS, 2,4,6-trinitrobenzenesulfonic acid.

Similarly, a positive effect of pregabalin has been demonstrated in IBS-D and mixed stool pattern IBS patients, but it did not lead to improvement in IBS-C patients. Although pregabalin could be beneficial for IBS abdominal pain, bloating and diarrhea, it did not improve quality of life, anxiety and depression (Saito et al., 2019). The different efficacy profile of pregabalin among IBS subtypes may be due to the low prevalence of visceral hypersensitivity in IBS-C. PD-217014 is a novel α_2_δ ligand with visceral analgesic activity that is potentially more potent than gabapentin and pregabalin. It has been shown to be effective in inhibiting the visceral hypersensitivity induced by an intra-colonic injection of 2,4,6-trinitrobenzene sulfonic acid in rats (Ohashi et al., 2008). However, a recent large-scale randomized clinical trial showed no significant efficacy of PD-217014 compared to placebo in reducing abdominal pain/discomfort in IBS patients (Houghton et al., 2025). These conflicting results on the efficacy of α_2_δ ligands in the IBS treatment need to be investigated by further studies on large samples, also to assess their safety profile.

Further potential drugs able to act through GABA modulation are topiramate, which acts on GABA-activated chloride channels, diazepam, by modulation of peripheral GABA-AR signaling, baclofen (i.e., a GABA-BR agonist), and CGP7930 (i.e., a GABA-BR positive allosteric modulator), currently used only in experimental settings (Loeza-Alcocer and Gold, 2022; Brusberg et al., 2009; Dudley et al., 2011; Nissen et al., 2018). Despite being a promising target for IBS treatment, activation of GABA-AR has been linked to serious adverse effects, including aggravation of severe colitis (Ma et al., 2018). Other alternative treatments for GABA-dependent GI symptoms are the use of probiotics, natural (e.g., *B. dentium* ATCC 27678) or genetically modified (e.g., *B. longum* with a plant derived GAD gene) GABA producers, or GABA containing functional foods (e.g., fermented goats’ milk and enriched bioactive yogurt) (Pokusaeva et al., 2017; Laroute et al., 2022; Jin et al., 2023; Gomes et al., 2025; García Mansilla et al., 2024; Minerv et al., 2009; Linares et al., 2016; Park et al., 2005; Ramos et al., 2025). Indeed, studies on ingested bacteria have shown that several probiotic bacterial genera have beneficial effects in relieving the symptoms of IBS patients (Chen et al., 2023). Despite some growing evidence, the efficacy of probiotics in IBS is limited in clinical practice by the uncertainty associated with the wide biological variability of bacterial strains (Moayyedi et al., 2010). Especially regarding bacteria producing GABA, evidence from clinical trial are really limited. In a recent randomized placebo-controlled clinical trial, the efficacy of administering *Bifidobacterium adolescentis* PRL 2019, a GABA-producing strain, was tested in a paediatric population with IBS according to Rome IV criteria. *Bifidobacterium adolescentis* PRL2019 was able to reduce the severity and frequency of IBS symptoms in children, with positive effect on bowel habits in patients with constipation-predominant IBS (IBS-C) (Giorgio et al., 2025).

Finally, in order to achieve beneficial effects at digestive level, another possible strategy may be direct GABA supplementation (Gros et al., 2021; Cataldo et al., 2020; Sokovic Bajic et al., 2019). Among GABA-based treatments, some are based on a combination of more compounds, such as the GABA combined with *M. officinalis* supplementation, that was able to reduce intestinal inflammation and oxidative stress. Furthermore, it was able to improve the IEB in a rat models of IBS. *Melissa officinalis* may enhance the therapeutic effects of GABA, either by inhibiting its metabolism *in vivo* or by directly influencing inflammation and nociception (Lucarini et al., 2024). Even though promising, also this combination has not yet been tested on IBS patients.

## 5 Discussion

The IBS pathophysiology is multifactorial and only partially understood (Bellini et al., 2014). However, genetic, immune, environmental, inflammatory, neurological and psychological factors may play a crucial role in MGB axis dysregulation leading to visceral hypersensitivity, intestinal epithelial barrier disruption, intestinal dysbiosis and changes in colonic motility (Bellini et al., 2014; Vasant et al., 2021). In recent years, emerging evidence has pointed out a possible involvement of GABAergic signaling pathways in the pathogenesis of IBS (Loeza-Alcocer et al., 2019; Gros et al., 2021; Cataldo et al., 2020; Sokovic Bajic et al., 2019). Indeed, GABA seems to be involved in the regulation of many GI functions, acting both at a central and peripheral level. Therefore, GABAergic dysregulation may exert different pathogenetic effects on different components of IBS depending on its site of action (CNS, peripheral or MGB axis).

At the CNS level, GABA seems to be involved and to modulate some crucial IBS aspects mainly linked to visceral sensitivity and mood disorders. Indeed, some areas of the CNS, such as the ACC, LHA and GABAergic projections from the former to the latter, may be altered by chronic abdominal pain and may play a role in symptom maintenance and the genesis of psychological comorbidities (Guo et al., 2024; Li et al., 2020; Juarez-Salinas et al., 2019). Supporting the close connection between these areas and IBS- psychological comorbidities, ACC is also a crucial hub for emotion and cognition, and it has been closely related to mood disorders associated with GI diseases (Guo et al., 2024). Indeed, inflammation and chronic GI pain have been demonstrated to be primary drivers that induce brain remodeling, especially in the ACC (Matisz and Gruber, 2022). In this regard, GABAergic neurons in ACC seem to play a pivotal role also in anxiety/depression like emotions and pain management (Guo et al., 2024).

In contrast, at the gut level, a GABAergic dysregulation seems to be mainly implicated in the development of dysmotility, alteration in cell proliferation (Hyland and Cryan, 2010), regulation of GI immune system (Aggarwal et al., 2018; Seifi et al., 2018) and epithelial barrier impairment (Sokovic Bajic et al., 2019). Additionally, at gut level, some studies have shown that eliciting GABAergic signaling may have a modulatory effect on the diversity of the gut-microbiota. GABA, thus, appears to be able to modulate various pathogenetic mechanisms of IBS, and a therapy that targets this system might be able to act on multiple IBS mechanisms simultaneously.

Therefore, in light of emerging evidence on the role of GABA, new therapies targeting GABAergic pathways may be reasonable. However, the majority of evidence regarding therapeutic approaches based on GABA to treat visceral pain, IBD-related abdominal pain or IBS has so far been evaluated only on animal models and therefore there are currently no approved or recommended therapies targeting the GABAergic system. In this regard, only the British Society of Gastroenterology guidelines for IBS mention pregabalin as a possible new therapy for visceral pain based on two randomized clinical trials, underlying however that further evidence is needed before considering this medication for IBS (Vasant et al., 2021). Drug acting on GABA pathways (e.g., gabapentin, pregabalin and PD-217014) have shown in few clinical trials to improve intestinal sensitivity and IBS symptoms, especially in patients with diarrhea (Houghton et al., 2007; Iturrino et al., 2014; Saito et al., 2019) and data are conflicting for other subtypes of IBS. Furthermore, many drugs were tested only on preclinical settings and not on IBS patients (Table 1). Therefore, there is a pressing need on new high-quality evidence from clinical trials supporting the interesting data emerged from the preclinical studies. Regarding the best target to modulate (e.g., GABA-AR vs. GABA-BR vs. other targets) there is currently no knowledge on this subject. It is plausible that GABAergic modulation mediated by GABA-B receptors may be more effective in modulating visceral hypersensitivity in IBS-D than in other subtypes of IBS, considering the known pathophysiological differences between the various subtypes of IBS.

On the other hand, another crucial source of GABA is the one derived from the gut-microbiota production or the diet-derived GABA. Indeed, several preclinical and clinical studies have demonstrated that GABA-producing bacteria in both viable and non-viable forms might represent a potential treatment for alleviating IBS symptoms, especially considering their effect on visceral hypersensitivity. However, also regarding these products, there are currently few clinical trials in human evaluating the effects of GABA-producing bacteria in IBS (Table 1).

Studies are also lacking regarding the difference in GABA supplementation through the diet and endogenous production of GABA. In this context, the recent study by Lucarini et al. on a rat model of post-inflammatory IBS, points out a possible encouraging therapeutic role of the supplementation of a combination of GABA and *M. officinalis* in treating IBS (Lucarini et al., 2024). However, this supplementation has not yet been tested in clinical practice.

These findings collectively support the notion that targeting GABAergic pathways could offer a promising therapeutic strategy for managing both the inflammatory and pain-related symptoms of IBS (Figure 1). The many available preclinical studies are essential because only through understanding the bacterial metabolism of GABA, the variability between strains, and the bioavailability of GABA will be possible to translate probiotic or postbiotic therapy to humans. Our hope is that in future we will have high-quality evidence supporting the effective usage of GABA-acting or GABA-based products to treat IBS.

In conclusion, emerging evidence underscores the important role of GABA in the pathophysiology of IBS, particularly in relation to visceral pain and the MGB axis dysregulation. A better understanding of the intricate relationships between GABAergic dysfunction, microbial influences, and IBS symptoms is essential for developing targeted therapies based on pathophysiological evidence. In this regard, continued research is warranted to bridge existing gaps in knowledge and enhance the clinical management of this complex disorder. Future randomized clinical trials involving GABA-based products are advisable to assess whether it may provide a new therapeutic chance for IBS patients.
